# Structural effects of asymmetric magnet shape on performance of surface permanent magnet synchronous motors

**DOI:** 10.1038/s41598-023-50366-z

**Published:** 2024-02-17

**Authors:** Jungwoo Choe, Hyuksung Kwon, Hyunwoo Kim, Doheon Koo, Hongyun So

**Affiliations:** 1 Department of Mechanical Engineering, Hanyang University, Seoul, 04763 South Korea; 2 Department of Electrical and Computer Engineering, Seoul National University, Seoul, 08826 South Korea; 3 Department of Electrical Engineering, Hanyang University, Seoul, 04763 South Korea; 4 Institute of Nano Science and Technology, Hanyang University, Seoul, 04763 South Korea

**Keywords:** Mechanical engineering, Electronic devices

## Abstract

This study proposes a novel surface permanent magnet synchronous motor (N-SPMSM) structure, which features asymmetric magnets attached to the rotor surface. The N-SPMSM structure exhibits reduced structural complexity and minimal electromagnetic performance degradation. The properties of N-SPMSM are analyzed by comparing its structural complexity (in terms of the shape) and manufacturing complexity and electromagnetic performance [in terms of the cogging–mutual torque ratio and back-electromotive force (EMF) values], with the corresponding values of a ring-type SPMSM (R-SPMSM) and step-skew-type SPMSM (T-SPMSM). The analysis results demonstrate that N-SPMSM has lower shape complexity than T-SPMSM and lower manufacturing complexity than both R-SPMSM and T-SPMSM. The cogging torque reduction and back-EMF performances of N-SPMSM are similar to that of R-SPMSM and T-SPMSM.

## Introduction

The twentieth century witnessed a rapid growth in the motor industry, resulting in similar advancements in the heavy-equipment, automotive, and home-appliance industry (covering home appliances such as refrigerators, washing machines, and computer peripherals). In the twenty-first century, the motor industry has emerged as a key module in advanced factory and logistics automation. This role extends to semiconductor equipment, display equipment, high-tech medical devices, and smart factories, as well as in small, high-precision–control applications such as robots, automated guided vehicles, autonomous vehicles, and drones^1^.

In particular, in the industrial-device sector that requires high speeds, there has been an increasing demand for high-speed precision motors that can directly operate various systems with minimal noise and maintenance, while providing high rotational energy and durability. An electric motor is a device that converts electrical energy into mechanical energy and consists of components such as stator, coil, rotor core, permanent magnet, bearing, housing, flange, bracket, and power cable. Electric motors are typically categorized into four types: brushless motors, brush motors, induction motors, and permanent magnet synchronous motors (PMSMs). Among these types, PMSMs are widely applied in the development of high-speed and high-efficiency motors owing to their ease of miniaturization and high torque density^2^. They have been evaluated to be the most suitable motor for high-speed applications, in terms of mechanical stability and power density^3,4^.

Depending on the type of permanent magnet attachment, PMSMs are classified into surface-PMSMs (SPMSMs), where the permanent magnets are attached to the surface of the rotor, and interior-PMSM (IPMSMs), where the permanent magnets are attached to the inside of the rotor. SPMSMs, in particular, have relatively simpler magnetic circuit designs and responsiveness, compared to that of IPMSMs. They exhibit linear torque–current and speed–voltage characteristics, making it easy to control the torque and speed with a relatively simple control algorithm^5–8^. However, SPMSMs have the disadvantage of noise and vibration generation because of the cogging torque, which makes precise speed control difficult. The cogging torque is a magnetic force generated by the interactions between the permanent magnet and stator steel when the rotor rotates during no-load operation, which causes magnetic field changes.

Research has been actively conducted on reducing the cogging torque of SPMSMs, focusing mainly on changing the shape of the rotor, pole of the stator, and number of slots. Anuja et al.^9^ studied the reduction in the cogging torque by optimizing the combination of pole pitch and arc. Xing et al.^10^ demonstrated a decrease in the cogging torque by skewing different pole-arc magnets. Yang et al.^11^ proposed a novel motor with the poles and slots by combining two types of motors with different poles–slots to reduce the cogging torque. Park et al.^12^ and Song et al.^13^ proposed a cycloid curved permanent magnet and an intersect magnet consequent pole structure, respectively. Although these studies proposed innovative designs, they significantly increased the complexity.

A significant portion of the research to date has focused on performance improvement at the expense of complexity increase^10,12^. Studies on reducing the complexity of motors, especially, those on reducing the structural complexity of SPMSMs, are lacking. This study presents a novel method to reduce the structural complexity of SPMSMs while minimizing the performance degradation due to cogging torque. In particular, this study proposes a novel SPMSM (N-SPMSM) structure using asymmetrically shaped permanent magnets.

The structural complexity, cogging-torque reduction, and back-electromotive force (EMF) performance are investigated through a characteristic analysis of the proposed N-SPMSM. The structural complexity of N-SPMSM is analyzed and compared to that of ring-type SPMSM (R-SPMSM) and step-skew–type SPMSM (T-SPMSM), in terms of the shape and manufacturing. The shape complexity is evaluated in terms of the number of surfaces to which the magnets are attached, and the manufacturing complexity considers the jig required for manufacturing. N-SPMSM's cogging-torque and back-EMF performances are verified through electromagnetic simulations using JMAG, a commercial software, and experiments using actual prototypes. The R-SPMSM and T-SPMSM are considered in the performance comparisons, and the cogging– mutual torque ratio and back-EMF values are selected as measurement indicators. The reminder of this paper is organized as follows. “Related works” section  describes the characteristics of SPMSMs and conventional rotors. “Analysis and design of N-SPMSM-I for design parameter” section  describes and analyzes the rotors of the proposed N-SPMSM, and “Performance evaluation” section analyzes the results of electromagnetic simulations and experimental evaluations of the N-SPMSM. Finally, “Conclusions” section describes the conclusions and future expectations.

## Related works

### Descriptions of the basic and proposed SPMSMs

**Figure 1:**
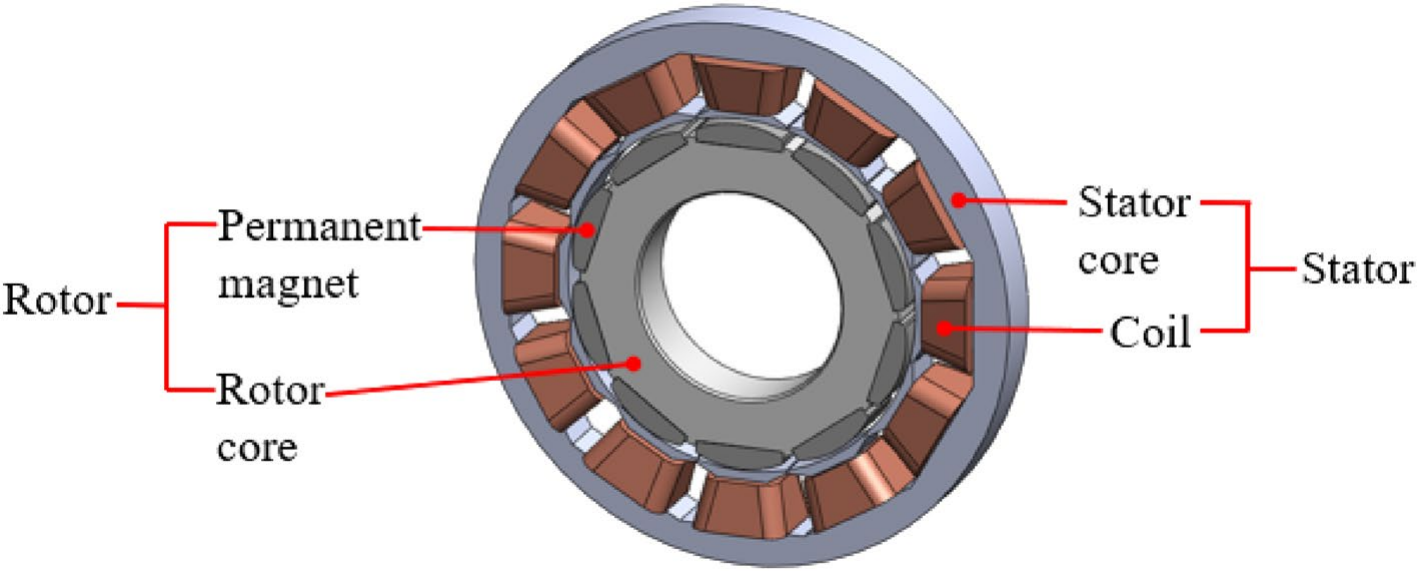
Structure of an SPMSM.

In general, SPMSMs consist of a stator and rotor, as depicted in Fig. 1. The stator comprises a stator core and coils, with the former providing support to the latter and serving as a path for the magnetic flux from the rotor's magnetic pole. The stator rotates the rotor by producing a magnetic field from the current flowing through the coil wound around the stator core. The stator core is fabricated by stacking materials with low coercivity, high permeability, less thickness, and high saturated magnetic-flux density, such as silicon steel plates or electrical sheets. The coil, which is a wire in the shape of a spiral, acts as an electromagnet that functions upon receiving current. The coil is made of copper or other materials with low electrical resistance and excellent electrical and thermal conductivity. In contrast, the rotor comprises both the rotor core and permanent magnets. The rotor converts the electrical energy received from the stator into mechanical energy and rotates. The rotor core is prepared from lightweight, durable, corrosion-resistant, and heat-resistant materials such as machined stainless steel and cast iron, and facilitates rotation. The permanent magnets are prepared in the form of rings or bars, by sintering neodymium materials, and provide magnetic flux.

This study focuses on six basic and two novel models of SPMSMs—symmetric-type SPMSM-I (S-SPMSM-I), symmetric-type SPMSM-II (S-SPMSM- II), ring-type SPMSM-I (R-SPMSM-I), ring-type SPMSM-II (R-SPMSM-II), step-skew–type SPMSM-I (T-SPMSM-I), step-skew–type SPMSM-II (T-SPMSM-II), novel-type SPMSM-I (N-SPMSM-I), and novel-type SPMSM-II (N-SPMSM-II), where SPMSM-I and SPMSM-II denote the small and large models, respectively. These models have been selected to investigate the structural complexity and electromagnetic characteristics of SPMSMs, in terms of the size and rotor type. Each model consists of a stator and rotor, with different types of rotors such as symmetric-type rotor (STR), ring-type rotor (RTR), and step-skew–type rotor (TTR). Stator-I and Stator-II are commonly used in SPMSM-I and SPMSM-II, respectively. The SPMSM types used in this study are summarized in Table 1.

**Table 1:** SPMSM types.

Type	Classification	Composition
Symmetric type	S-SPMSM-I	Stator-I + STR-I
	S-SPMSM-II	Stator-II + STR-II
Ring type	R-SPMSM-I	Stator-I + RTR-I
	R-SPMSM-II	Stator-II + RTR-II
Step-skew type	T-SPMSM-I	Stator-I + TTR-I
	T-SPMSM-II	Stator-II + TTR-II
Novel type	N-SPMSM-I	Stator-I + NTR-I
	N-SPMSM-II	Stator-II + NTR-II

**Figure 2:**
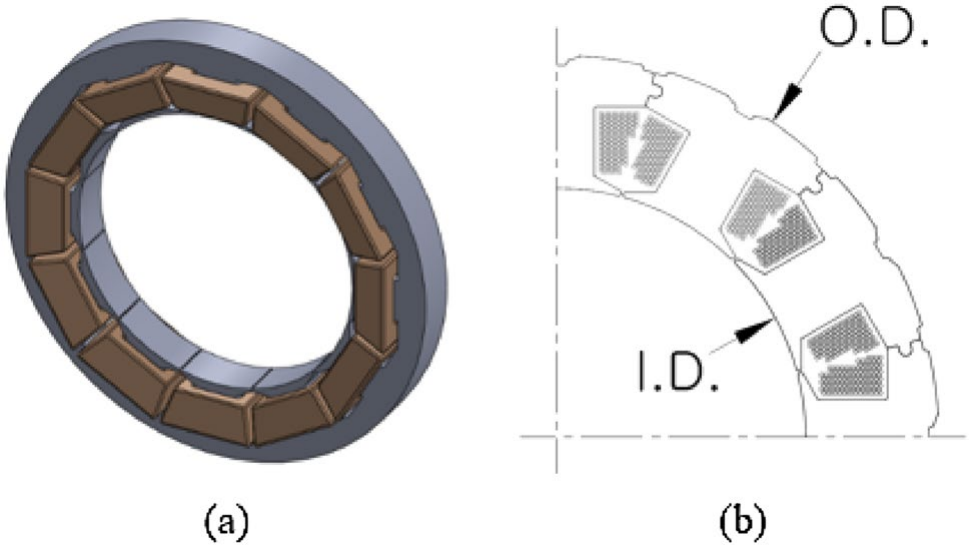
Structure of the stator: (**a**) shape of the stator and (**b**) quarter cross-section of the stator.

Figure 2 and Table 2 show the shapes and basic specifications of Stator-I and Stator-II. Figure 2a illustrates the shape of the stator and Fig. 2b shows a quarter of the cross-section of the stator. O.D and I.D represent the outer diameter and inner diameter, respectively, of the stator. In Table 2, the supply voltage is the same (28 V_dc_) for both Stator-I and Stator-II, whereas the supply current is 4.5 A_rms_ and 6.5 A_rms_ for Stator-I and Stator-II, respectively. In terms of size, Stator-I has an O.D. of 62.0 mm, I.D. of 40.4 mm, and length of 6 mm. It is smaller than Stator-II, which has O.D. of 70.2 mm, I.D. of 66.3 mm, and length of 8.5 mm. Both stators have the same number of slots and are of the same material grade.

**Table 2:** Specifications of the stators.

Item	Unit	Stator-I	Stator-II
Supply voltage	V_dc_	28	28
Supply current	A_rms_	4.5	6.5
Number of slots	–	12	12
O.D.	mm	62.0	70.2
I.D.	mm	40.4	66.3
Length	mm	6	8.5
Material grade	–	S8	S8

**Figure 3:**
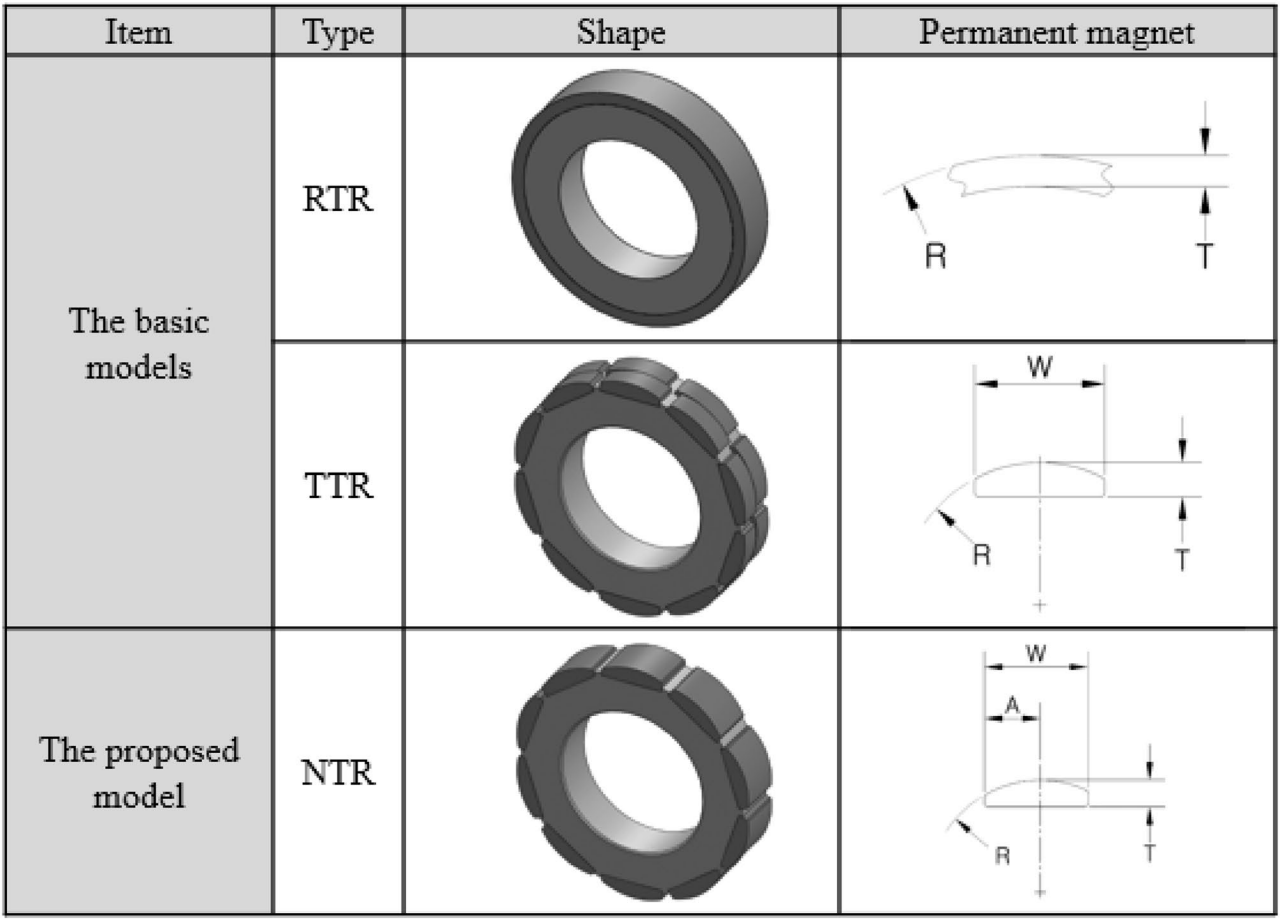
Shapes of the rotors.

Figure 3 and Table 3 show the shapes and specifications, respectively, of the rotors. The dimensions of the permanent magnets are presented in Table 4. Figure 3 illustrates that the permanent magnets are bonded to the rotor core; the cross-section of the permanent magnet is also presented. Figure 3 and Table 4 indicate that the permanent magnets used in RTR and TTR are symmetrical with respect to the center line. The permanent magnet used in NTR is asymmetrical with respect to the center line. The ring permanent magnet produces a relatively small magnetic flux compared to that of the segmented permanent magnets.

**Table 3:** Design specifications of the rotors.

Item	Unit	Type-I			Type-II		
		RTR	TTR	NTR	RTR	TTR	NTR
Total PM volume	mm^3^	1720	1640	1630	2886	2720	2710
Remanence	T	1.18	1.32	1.32	1.18	1.32	1.32
Coercivity	kA/m	891	979	979	891	979	979
Rotor O.D.	mm	35.4	37.0	37.0	34.9	36.3	36.3
Intrinsic coercivity	kA/m	1273	1592	1592	1273	1592	1592
Energy product	kJ/m^3^	286	354	354	286	354	354

**Table 4:** Dimensions of the permanent magnets.

Item	Unit	RTR-I	RTR-II	TTR-I	TTR-II	NTR-I	NTR-II
Radius, *R*	mm	19.75	20	10.9	11.7	10.9	11.65
Thickness, *T*	mm	2	2.5	2.6	3.2	2.6	3.2
Length, *L*	mm	7.4	9.8	3.7	4.9	7.4	9.8
Skew angle	°	3	3	3	3	3	3
Width, *W*	mm	–	–	10.1	9.8	10.1	9.8

### Electromagnetic properties of SPMSMs

Unlike other motors, PMSMs suffer from the cogging torque generated by the reluctance difference between the permanent magnet of the rotor and steel of the stator. Because such cogging torque has a significant influence on the noise and vibration generation in PMSMs, it must be reduced as much as possible during the design. The cogging torque (${T}_{cog}$) can be expressed as (1)^14^:

1 $$T_{cog} (\alpha ) = - \frac{{\partial W_{m} (\alpha )}}{\partial \alpha }$$

where *α* is the position angle of the rotor and *W*_*m*_ is the magnetic energy of SPMSM. The magnetic energy of SPMSM can be expressed as (2):

2 $$\begin{aligned} W_{m} (\alpha ) & = \frac{1}{{2\mu_{0} }}\int_{V}^{{}} {B^{2} dV} \\ & = \frac{1}{{4\mu_{0} }}\left( {R_{2}^{2} - R_{1}^{2} } \right)\int_{0}^{{L_{stk} }} {\int_{0}^{2\pi } {G^{2} (\theta ,\,\,z)B^{2} (\theta ,\,\,\alpha )d\theta dz} } \\ \end{aligned}$$

where *μ*_0_ is the permeability of air, *R*_2_ is the outer radius of airgap, *R*_1_ is the inner radius of air gap, *L*_*stk*_ is the axial length of SPMSM, *G* is the airgap relative permeance of SPMSM, B is the airgap flux density in an equivalent slotless SPMSM, *θ* is the angle, and *z* is the axial position of SPMSM.

If the slots are not skewed, *G*^2^ can be expressed by the Fourier series as follows^15^:

3 $$G^{2} (\theta ) = G_{a0} + \sum\limits_{k = 1}^{\infty } {G_{ak} \cos kN_{s} \theta }$$

where *G*_*a0*_ and *G*_*ak*_ are the Fourier series coefficients, and *N*_*s*_ is the number of slots.

Furthermore, if the magnet shapes of N-pole and S-pole are same, *B*^2^ is can be expressed by the Fourier series as follows:

4 $$B^{2} (\theta ) = B_{a0} + \sum\limits_{m = 1}^{\infty } {B_{am} \cos mN_{p} \left( {\theta + \alpha } \right)}$$

where *B*_*a0*_, and *B*_*am*_ are the Fourier series coefficients, and *N*_*p*_ is the number of poles.

Substituting (3) and (4) to (1) and (2), the cogging torque of PMSM can be expressed as (5):

5 $$T_{cog} (\alpha ) = - \frac{{\pi L_{stk} }}{{4\mu_{0} }}\left( {R_{2}^{2} - R_{1}^{2} } \right)\sum\limits_{n = 1}^{\infty } {nN_{L} G_{ak} B_{am} \sin nN_{L} \alpha }$$

where *N*_*L*_ is the least common multiple between the slot number (*N*_*s*_) and the pole number (*N*_*p*_), and the harmonic order of *G*^*2*^ and *B*^*2*^ must be satisfied as follow condition^16^:

6 $$kN_{s} = mN_{p} = nN_{L}$$

From (5), the cogging torque is determined by the harmonics of airgap relative permeance and airgap flux density. If the stator shape is same, the cogging torque is determined by the harmonic of airgap flux density in an equivalent slotless machine since the harmonic of airgap relative permeance is same. This means that the cogging torque can be decreased by changing the permanent magnet shape and arrangement, as the airgap flux are related to the permanent magnet shape and arrangement.

The mutual torque (${T}_{m}$) is the torque produced by the interaction of the magnetic fields produced by permanent magnets and winding currents, and is given by (7):

7 $$T_{m} = Ni\frac{d\phi }{{d\theta }}$$

where $\mathrm{\varnothing }$, $N$, $\theta$, and $i$ are the flux linking the single-turn coil, number of turns of a coil, rotor mechanical angle, and current of the coil, respectively^17^. Equation (7) indicates that the PMSM exhibits a higher torque when the strength of the magnet, number of turns of the coil, or current strength increases. The strength of the permanent magnet is determined by its size and material, the number of turns is limited by the motor driving speed, and the current strength is limited by the heat capacity.

### Principle of cogging-torque reduction in novel-type rotor

The principle of cogging-torque reduction in N-SPMSM is explained through electromagnetic simulations. S-SPMSM-I, right-side–type SPMSM-I (G-SPMSM-I), and left-side–type SPMSM-I (L-SPMSM-I) models are used to analyze the principle of cogging-torque reduction in N-SPMSMs. Here, S-SPMSM-I, G-SPMSM-I, and L-SPMSM-I consist of Stator-I and symmetric-type rotor (STR-I), Stator-I and right-side rotor-I (GTR-I), and Stator-I and left-side rotor-I (LTR-I), respectively. Table 5 lists the configurations of G-SPMSM-I and L-SPMSM-I. Here, Stator-I means the stator commonly used in the basic models.

**Table 5:** G-SPMSM and L-SPMSM types.

Type	Classification	Composition
Right-side type	G-SPMSM-I	Stator-I + GTR-I
Left-side type	L-SPMSM-I	Stator-I + RTR-I

**Figure 4:**
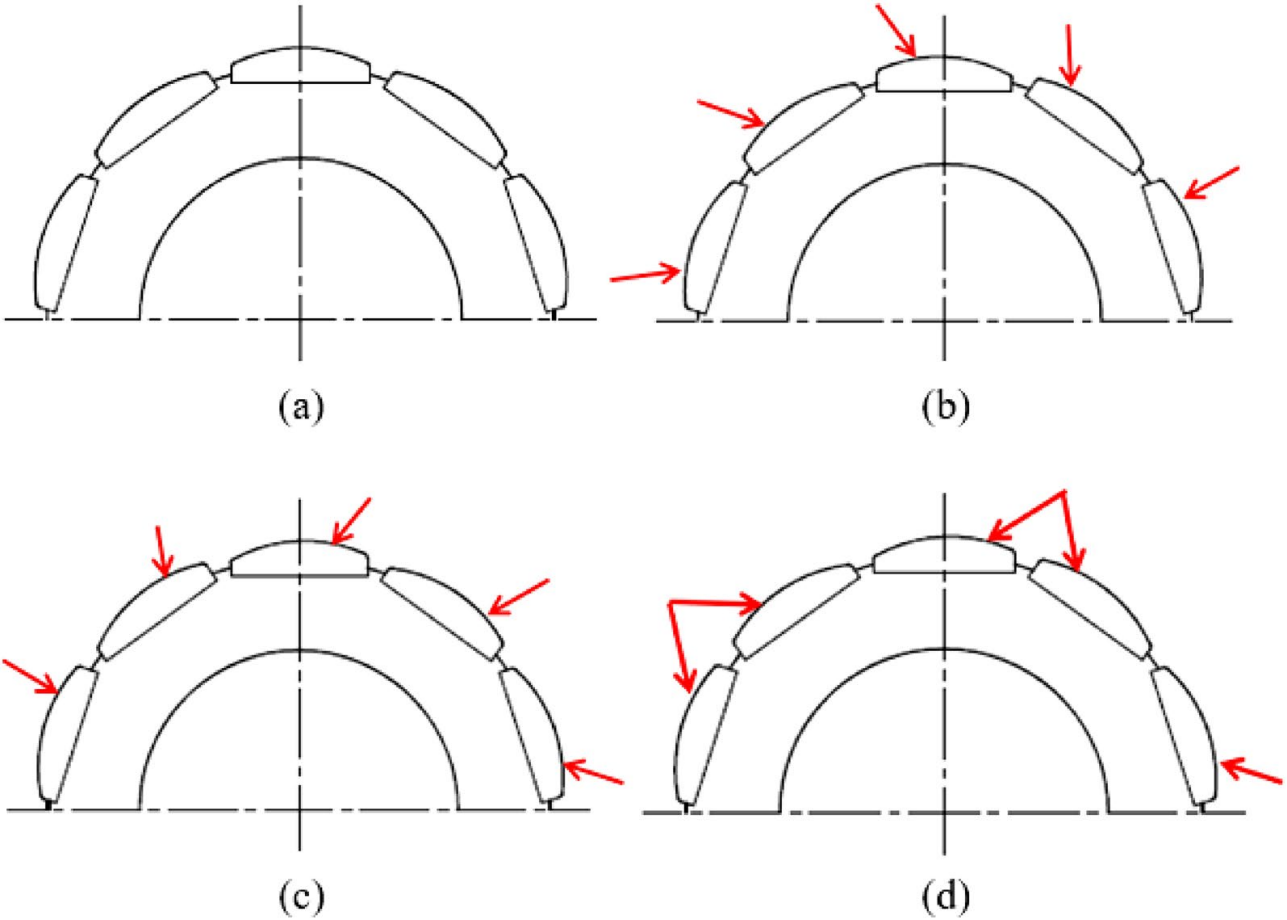
Half-sections of the models used to analyze the principle of cogging-torque reduction: (**a**) STR-I, (**b**) GTR-I, (**c**) LTR-I, and (**d**) NTR-I.

Figure 4 shows a half-section of the models used to analyze the principle of cogging-torque reduction; Fig. 4a represents the model in which symmetric permanent magnets are used and Fig. 4d represents the NTR-I model. Figure 4b,c show the half-sections of GTR-I and LTR-I, respectively; the rotor core and permanent magnet are similar to ones used in NTR-I. Based on the centerline of each magnet, permanent magnets are arranged such that the thick side of the magnet is to the right in GTR-I, whereas it is to the left in LTR-I; in Fig. 4, the arrows identify the thick side of the permanent magnet.

As the cogging torque changes according to the shape and arrangement of the magnets, the cogging torques of S-SPMSM-I, G-SPMSM-I, L-SPMSM-I, and N-SPMSM-I are analyzed using the JMAG electromagnetic analysis program. In the JMAG analysis, the material parameters are set as: copper for the coil, 35JN230 for the stator core, NMX-S45H for the permanent magnet, and S45C for the rotor core.

**Figure 5:**
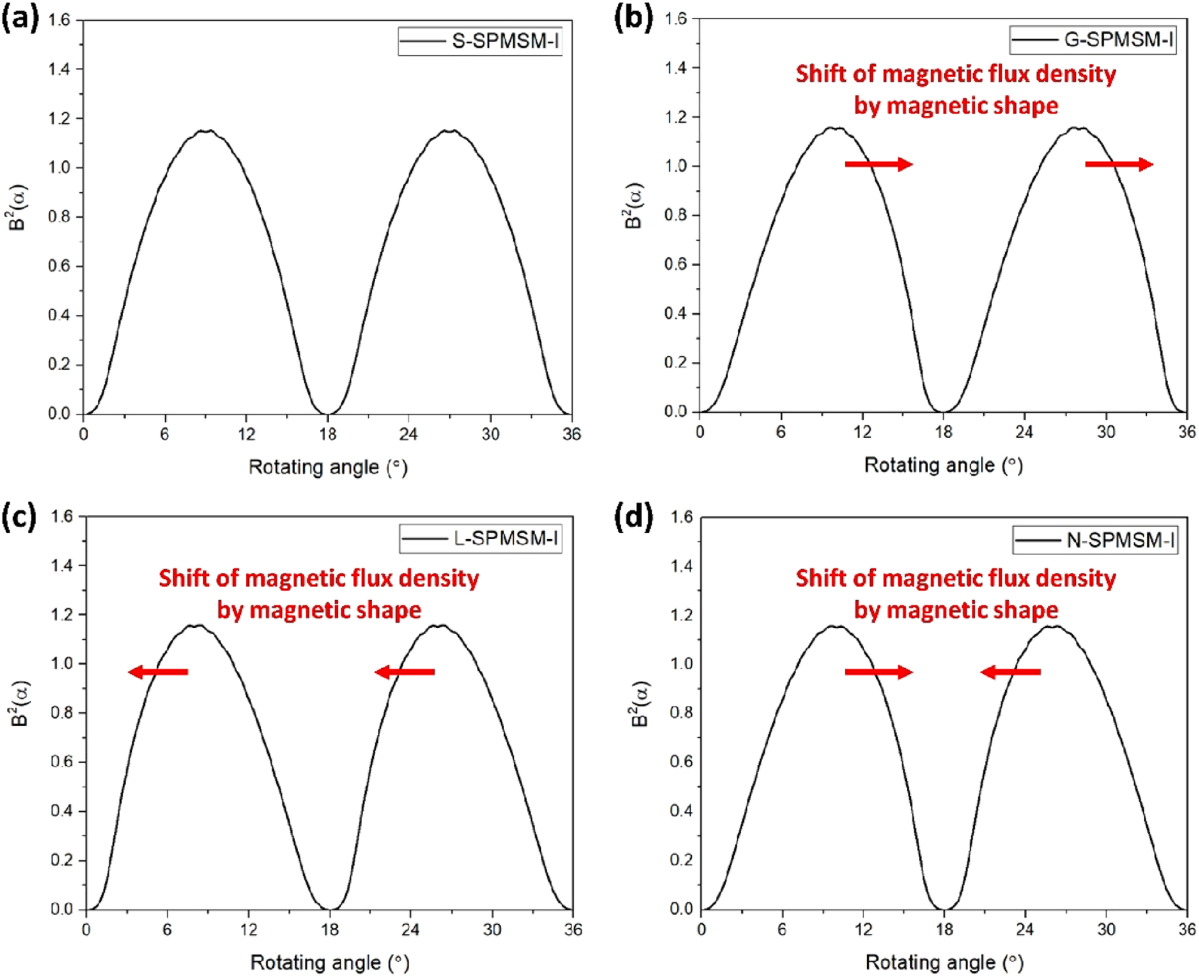
Simulation results of airgap magnetic flux density in the equivalent slotless SPMSM-I models (**a**) S-SPMSM-I, (**b**) G-SPMSM-I, (**c**) L-SPMSM-I, and (**d**) N-SPMSM-I.

**Figure 6:**
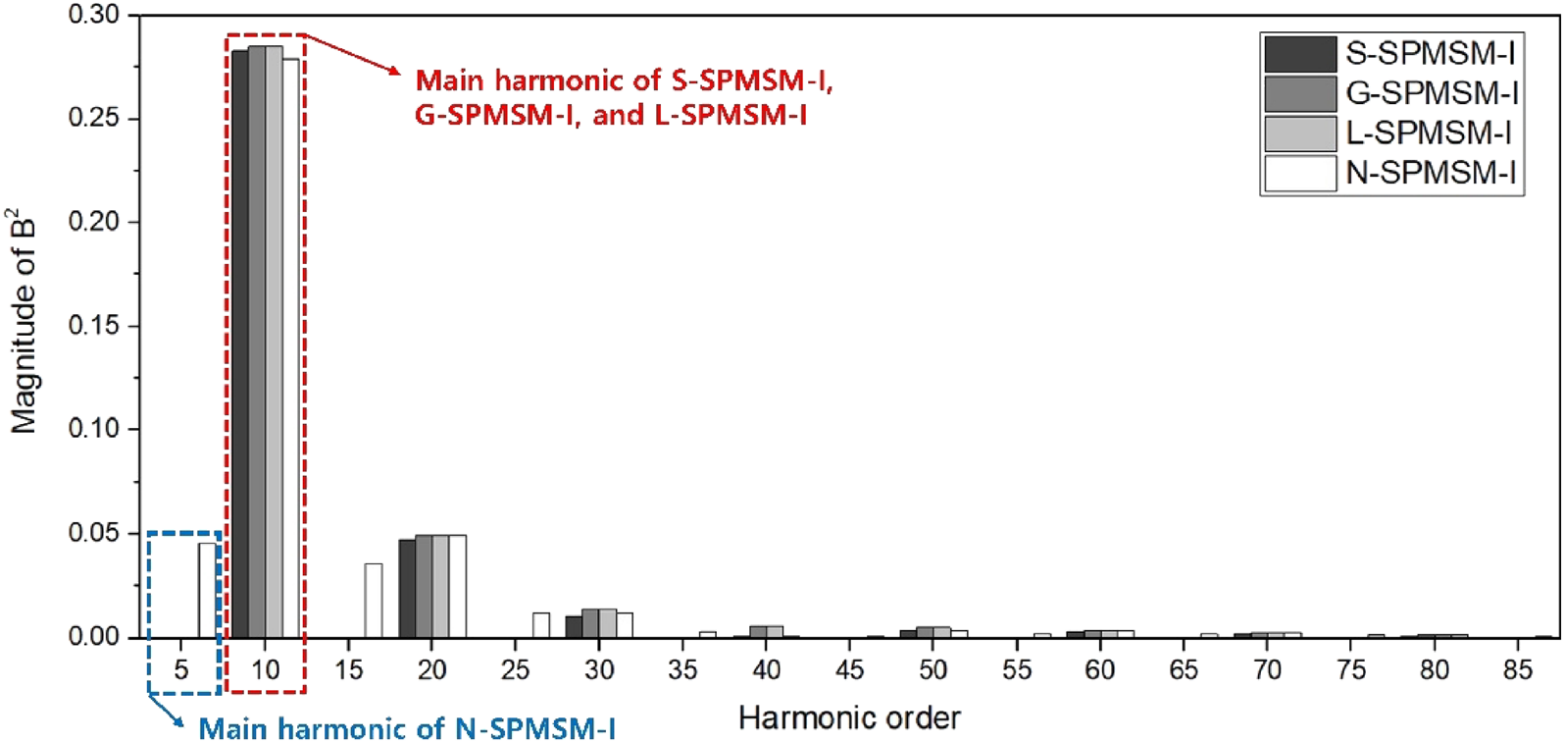
Harmonic FFT results of airgap magnetic flux density.

From (1), the cogging torque is determined by the harmonic of airgap magnetic flux density in the equivalent slotless PMSM under the same condition of stator shape. Figure 5 shows the the airgap magnetic flux density (*B*^*2*^) for the models as shown Fig. 4. Furthermore, Fig. 6 shows FFT results of the airgap magnetic flux density (*B*^*2*^). Since the magnetic shapes of N-pole and S-pole are same in S-SPMSM-I, G-SPMSM-I, and L-SPMSM-I, the period of airgap magnetic flux density is 2*π*/*N*_*p*_. However, the magnetic shapes of N-pole and S-pole are different in N-SPMSM-I, the period of airgap magnetic flux density is twice that of other models as shown Fig. 6. Therefore, the distribution of *B*^*2*^ can be expressed as follows:

8 $$B^{2} (\theta ) = B_{a0} + \sum\limits_{m = 1}^{\infty } {B_{am} \cos m\frac{{N_{p} }}{2}\left( {\theta + \alpha } \right)}$$

Substituting (3) and (8) to (1) and (2), the cogging torque of PMSM can be expressed as (9):

9 $$T_{cog} (\alpha ) = - \frac{{\pi L_{stk} }}{{4\mu_{0} }}\left( {R_{2}^{2} - R_{1}^{2} } \right)\sum\limits_{n = 1}^{\infty } {nN_{L} G_{ak} B_{am} \sin nN_{L} \alpha }$$

where *N*_*L*_ is the least common multiple between the slot number (*N*_*s*_) and the pole pair number (*N*_*p*_/2), and the harmonic order of *G*^*2*^ and *B*^*2*^ must be satisfied as follow condition:

10 $$kN_{s} = m\frac{{N_{p} }}{2} = nN_{L}$$

From (10), the harmonic order of *B*^*2*^ that determines the cogging torque is twice that of other models. Table 6 shows the harmonic order and magnitude of airgap magnetic flux density (*B*^*2*^) that determines the cogging torque. From Table 6, the cogging torque of N-SPMSM-I is lower than that of other models. Therefore, the cogging torque of N-SPMSM-I can be reduced compared to other models.

**Table 6:** Harmonic analysis of airgap magnetic flux density (*B*^*2*^) that determines the main cogging torque.

Content	S-SPMSM-I	G-SPMSM-I	L-SPMSM-I	N-SPMSM-I
Harmonic order of *B*^*2*^	6	6	6	12
Magnitude of *B*^*2*^	0.0032	0.00235	0.00236	0.000228

**Figure 7:**
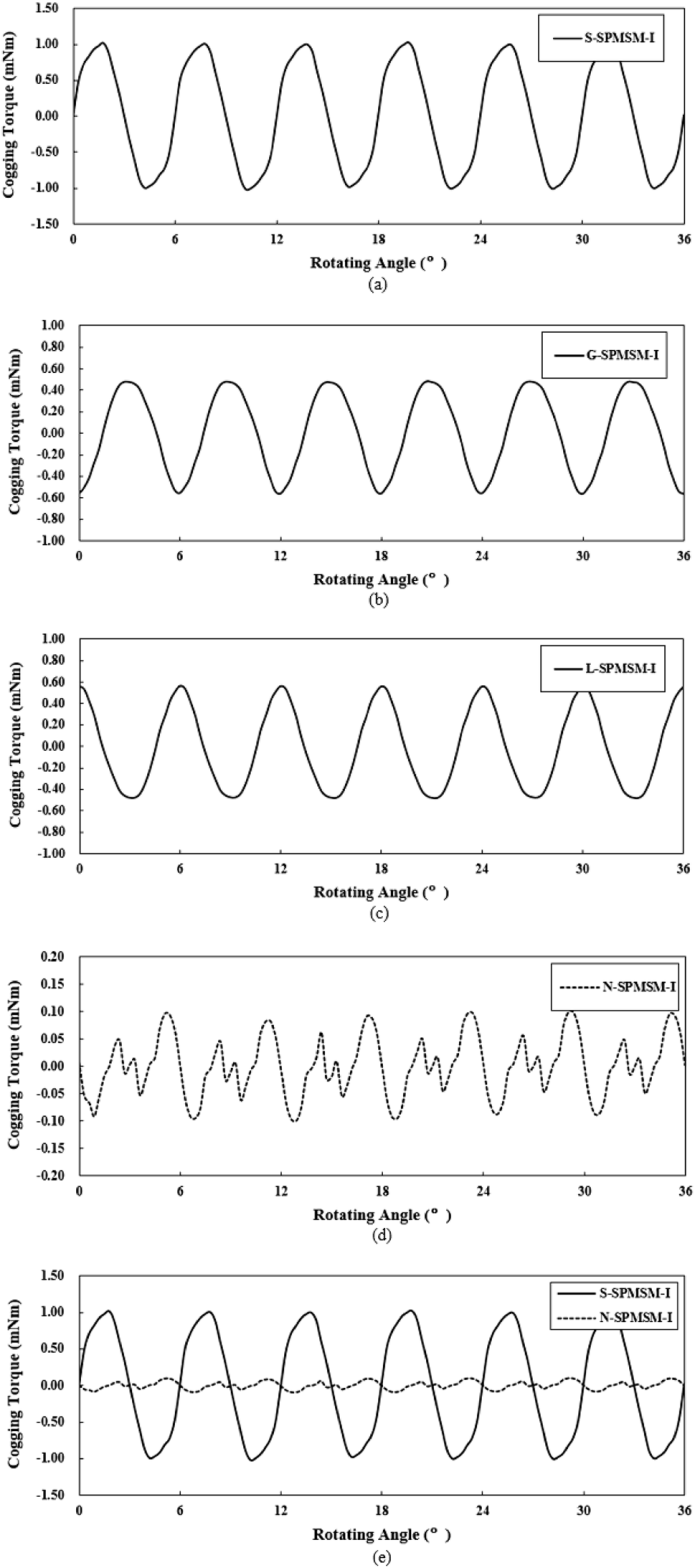
Simulation results of cogging torques of the SPMSM-I models for analysis of cogging-torque reduction principle: (**a**) S-SPMSM-I, (**b**) G-SPMSM-I, (**c**) L-SPMSM-I, (**d**) N-SPMSM-I, and (**e**) overlap between N-SPMSM-I and S-SPMSM-I.

Figure 7 shows the simulation results of the cogging torque of the SPMSM-I models for the analysis of the cogging-torque reduction principle. Figure 7a–d are cogging-torque graphs of the S-SPMSM-I, G-SPMSM-I, L-SPMSM-I, and N-SPMSM-I, respectively. In Fig. 7b,c, it is noted that, if the shape of the magnet is arranged on the left or right side, the cogging-torque graph also moves in the direction of the rotation-angle axis. In addition, the amplitude of the final graph can be reduced by overlapping the graphs of G-SPMSM-I and L-SPMSM-I, because of the phase differences between the two graphs. These analysis results imply that a rotor structure that provides low cogging torque is possible by combining the magnet arrangements of G-SPMSM-I and L-SPMSM-I. N-SPMSM-I was proposed based on the above principle, and in Fig. 7a,d,e, the cogging-torque peak-to-peak value of N-SPMSM-I is 0.2 mNm, which is 90% less than the cogging-torque peak-to-peak value of S-SPMSM-I. The torque ripple is determined by the cogging torque and back-EMF. By the principle of energy conversion, the magnetic torque can be expressed as follows.

11 $$T = \frac{{e_{a} i_{a} + e_{b} i_{b} + e_{c} i_{c} }}{{\omega_{m} }} = \frac{{3E_{1} I_{m} }}{{2\omega_{m} }} + \frac{3}{2}\sum\limits_{n = 6k} {\frac{{\left( { - E_{n - 1} + E_{n + 1} } \right)I_{m} }}{{\omega_{m} }}\cos n\omega t}$$

where *e*_*a*_, *e*_*b*_, and *e*_*c*_ are the back-EMF of each phase, *ω*_*m*_ is the mechanical angular speed, *E*_*1*_ is the 1st harmonic of back-EMF, *I*_*m*_ is the magnitude of current, *E*_n-1_ is (*n*-1)th harmonic of back-EMF, *E*_*n*+1_ is (*n* + 1)th harmonic of back-EMF, and *n* is harmonic order.

**Figure 8:**
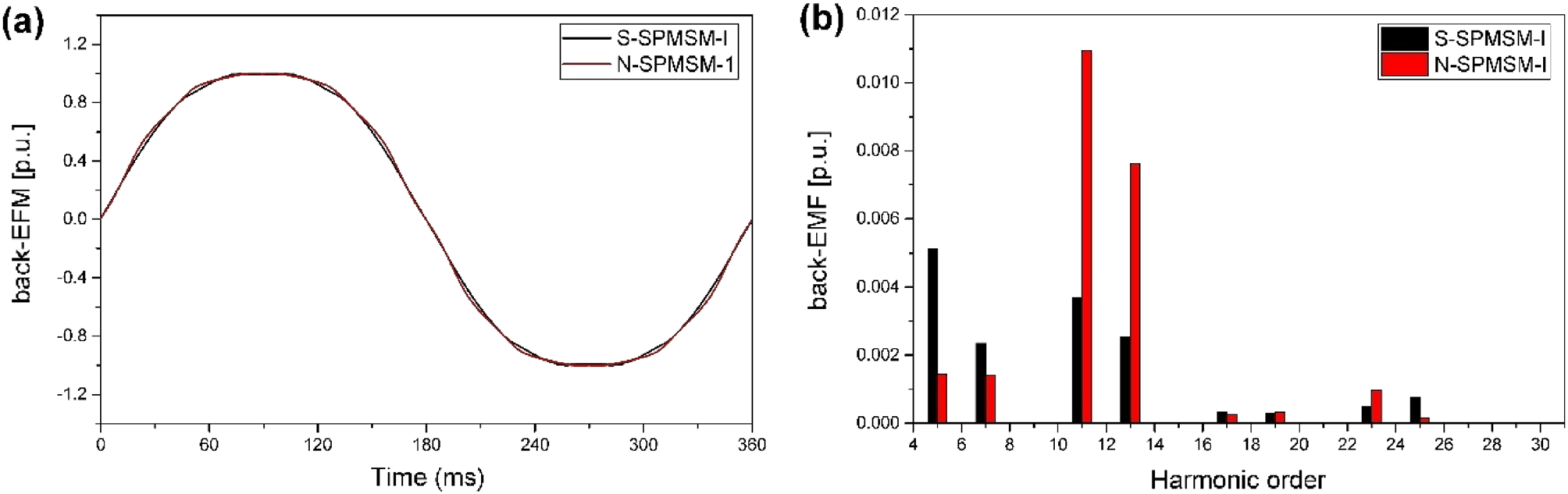
Normalized back-EMF of S-SPMSM-I and N-SPMSM-I (**a**) waveform (**b**) FFT result.

**Figure 9:**
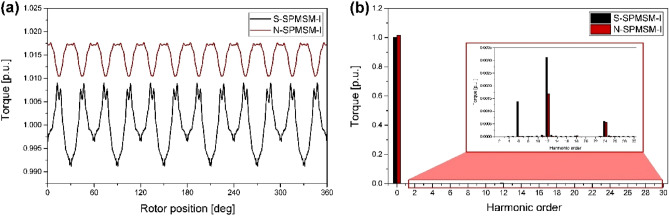
Normalized magnetic torque of S-SPMSM-I and N-SPMSM-I (**a**) torque waveform (**b**) FFT result.

Figure 8 shows the back-EMF of S-SPMSM-I and N-SPMSM-I. In Fig. 8a, the waveform of back-EMF is similar. However, the harmonic component of back-EMF is different in Fig. 8b. The main harmonic component of back-EMF in S-SPMSM-I is 5th and 7th order, but the main harmonic component of back-EMF in N-SPMSM-I is 11th and 13th order. This means the main harmonic component of torque ripple in S-SPMSM-I is 6th order, and in N-SPMSM-I is 12th order. From (11), the magnetic torque can be calculated as shown Fig. 9. As the torque is analyzed, the torque ripple of S-SPMSM-I is 6th order, and the torque ripple of N-SPMSM﻿-I is 12th order. From Fig. 9, the torque ripple of N-SPMSM﻿-I is lower than that of S-SPMSM-I. Therefore, the proposed magnet shape can reduce the cogging torque and torque ripple.

### Analysis and design of N-SPMSM-I for design parameter

**Figure 10:**
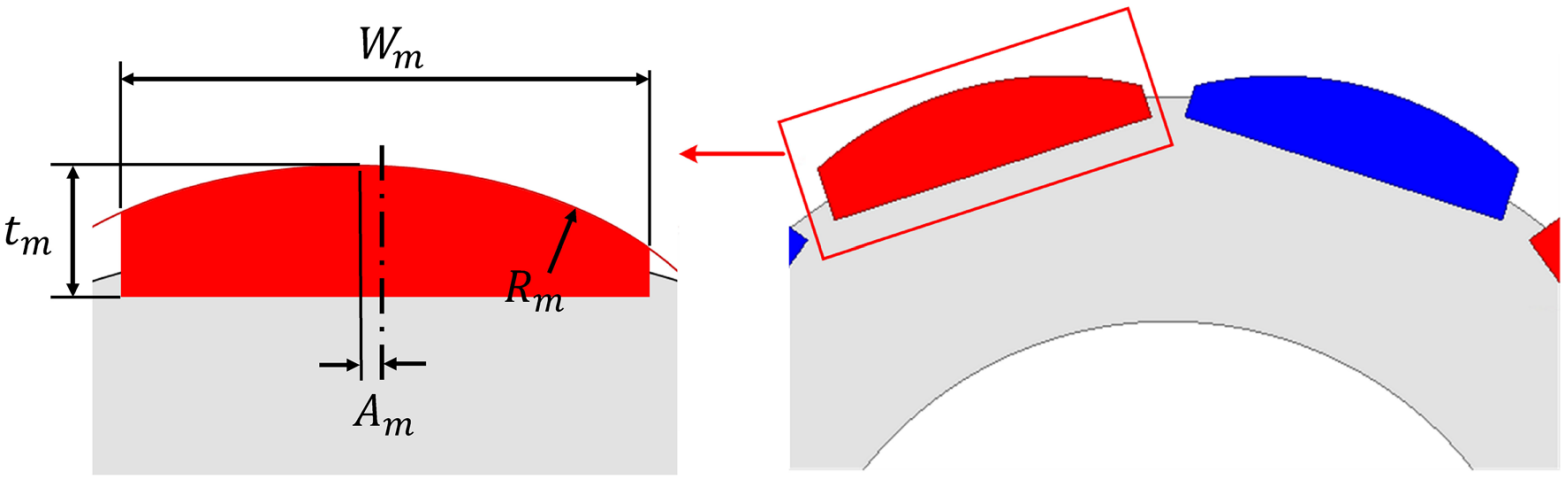
Design parameter of N-SPMSM-I.

**Figure 11:**
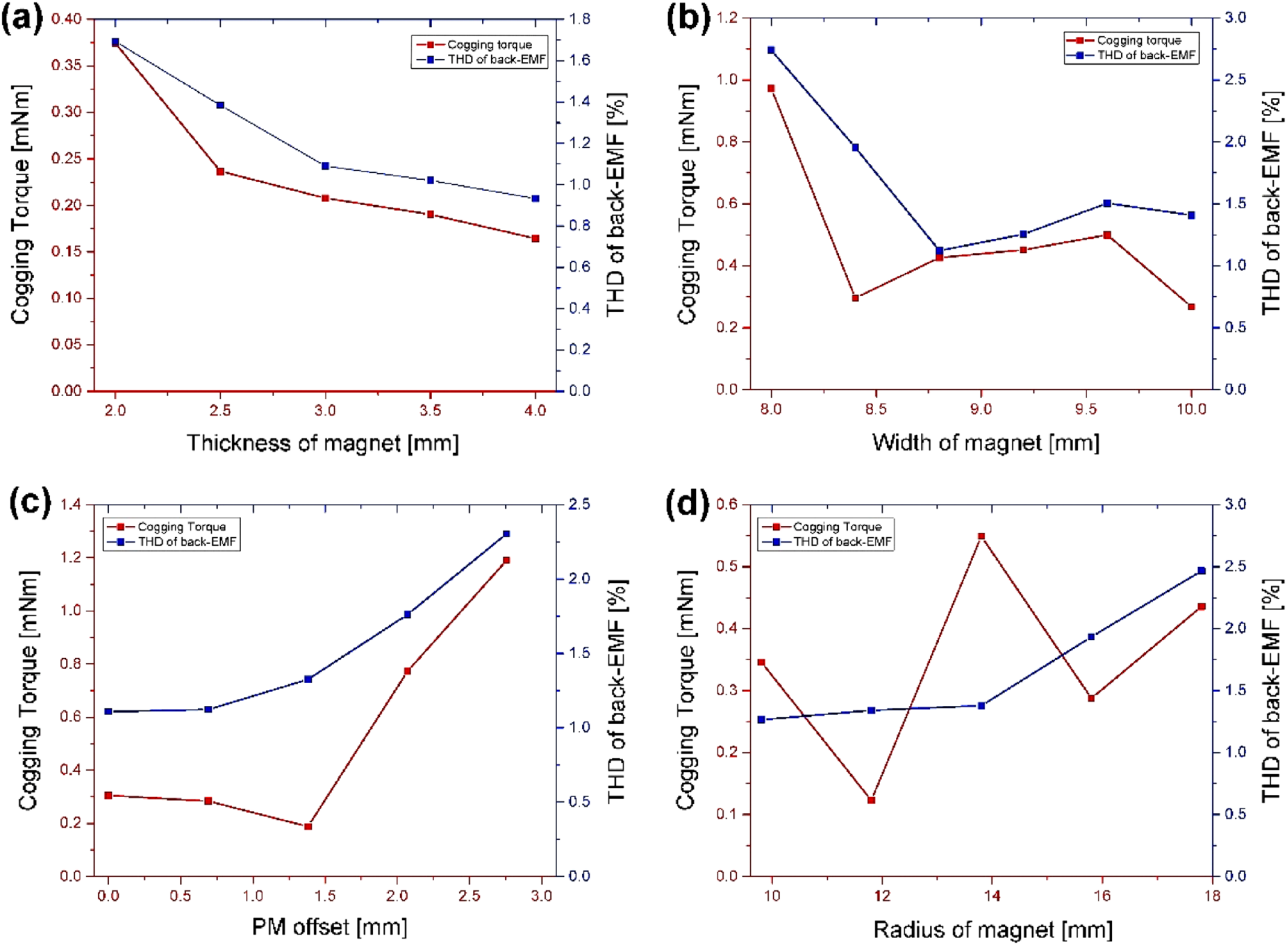
Analysis for design parameter of N-SPMSM-I (**a**) thickness of magnet (**b**) width of magnet (**c**) PM offset (**d**) radius of magnet.

**Figure 12:**
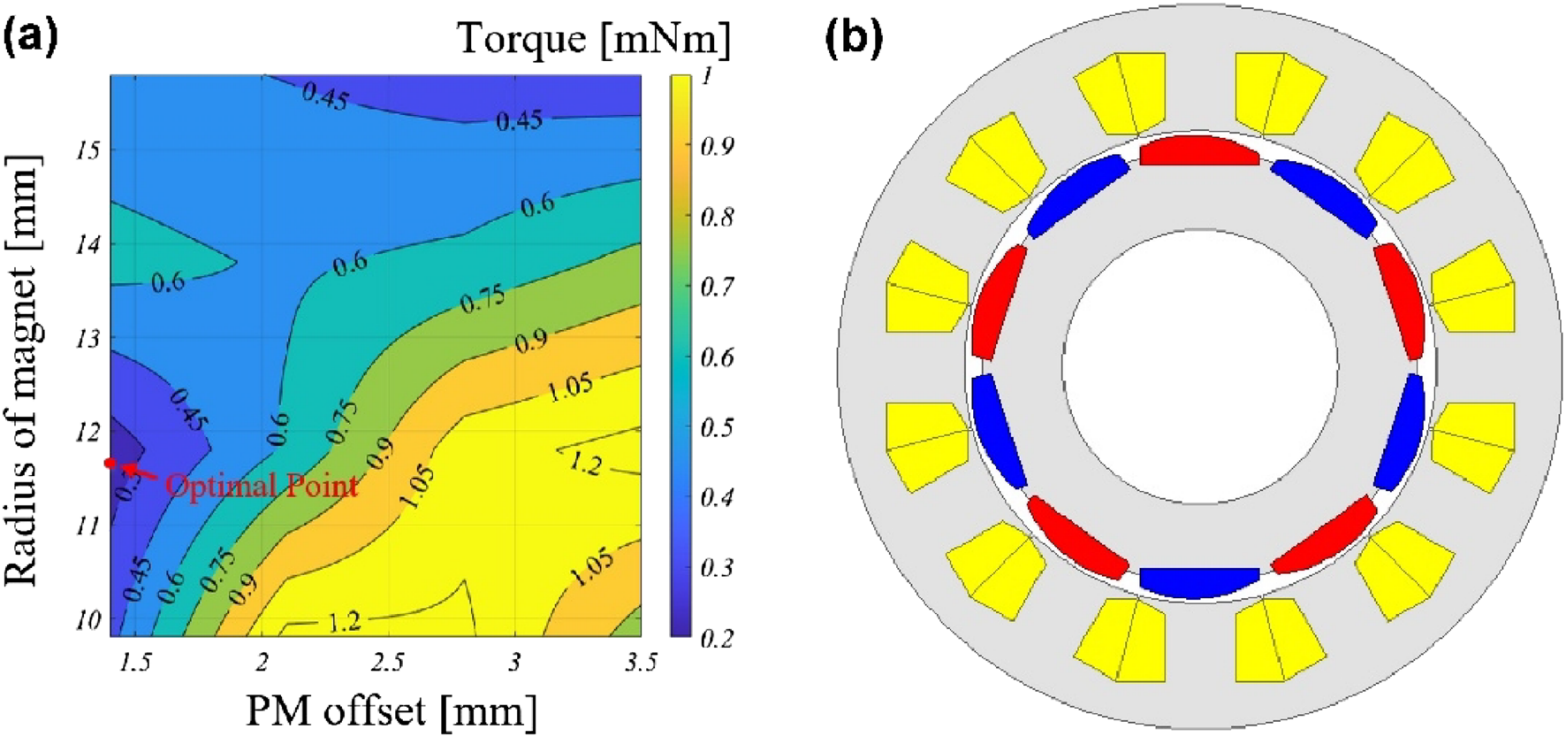
Optimal design (**a**) cogging torque map (**b**) optimal design model.

In “Principle of cogging-torque reduction in novel-type rotor” section, the effectiveness of N-SPMSM-I is discussed. In this chapter, the analysis and design of N-SPMSM-I for design parameter. Figure 10 shows the design parameters of N-SPMSM-I. In Fig. 10, the width of magnet is defined as *W*_*m*_, the radius of magnet is defined as *R*_*m*_, permanent magnet (PM) offset is defined as *A*_*m*_, and thickness of magnet is defined as *t*_*m*_. Figure 11 shows the analysis result for four design parameters. As thickness of magnet is increased, the cogging torque and total harmonic distortion (THD) of back-EMF is reduced in Fig. 11a. In Fig. 11b, the cogging torque and THD of back-EMF is analyzed for width of magnet. Considering the cogging torque and THD of back-EMF, there is the optimal design point. However, the thickness and width of magnet are usage of magnet. There is the usage of magnet determines the cost of motor. Therefore, the thickness and width of magnet must be reduced. Considering the performance such as the cogging torque, THD of back-EMF, and magnetic torque, the thickness and width are designed as 2.6 mm and 10.1 mm, respectively. Figure 11c shows the analysis results for PM offset. As the PM offset is increased, the THD of back-EMF is increased. However, the cogging torque for PM offset has the optimal design point. Figure 11d shows the analysis results for radius of magnet. As the radius of magnet is increased, THD of back-EMF is increased. The cogging torque is sensitive for the radius of magnet. Figure 12 shows the optimal design for reducing cogging torque. From Fig. 12a, which is the cogging torque map for design parameters, the radius of magnet and PM offset are designed as 11.8 mm and 1.4 mm, respectively. Figure 12b shows the optimal design result, and the cogging torque of optimal model is 0.22 mNm.

### Structural complexity of the rotors

**Figure 13:**
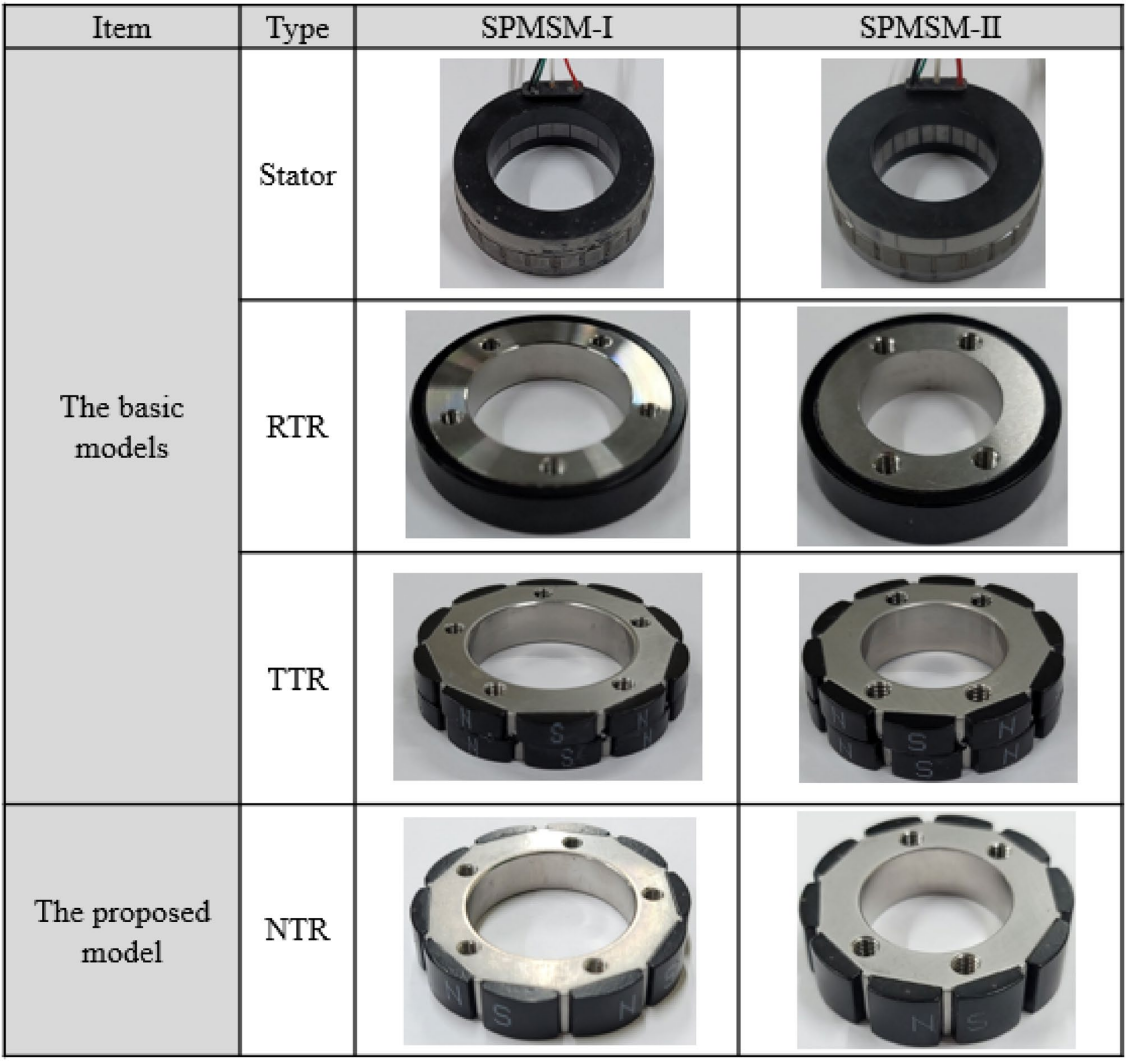
Prototypes of the SPMSMs.

Next, we analyze the structural complexities of the basic models and the proposed model. The structural complexity of each rotor is analyzed from the shape and manufacturing perspectives. The shape and manufacturing process of the rotor are major factors that affect the structural complexity^18^. The shape complexity is evaluated as the number of surfaces required for permanent-magnet attachment per pole, and the manufacturing complexity is evaluated by the need for additional manufacturing jigs. Figure 13 shows the prototypes of the SPMSMs. The exteriors of the stators are molded and show power and ground wires.

**Figure 14:**
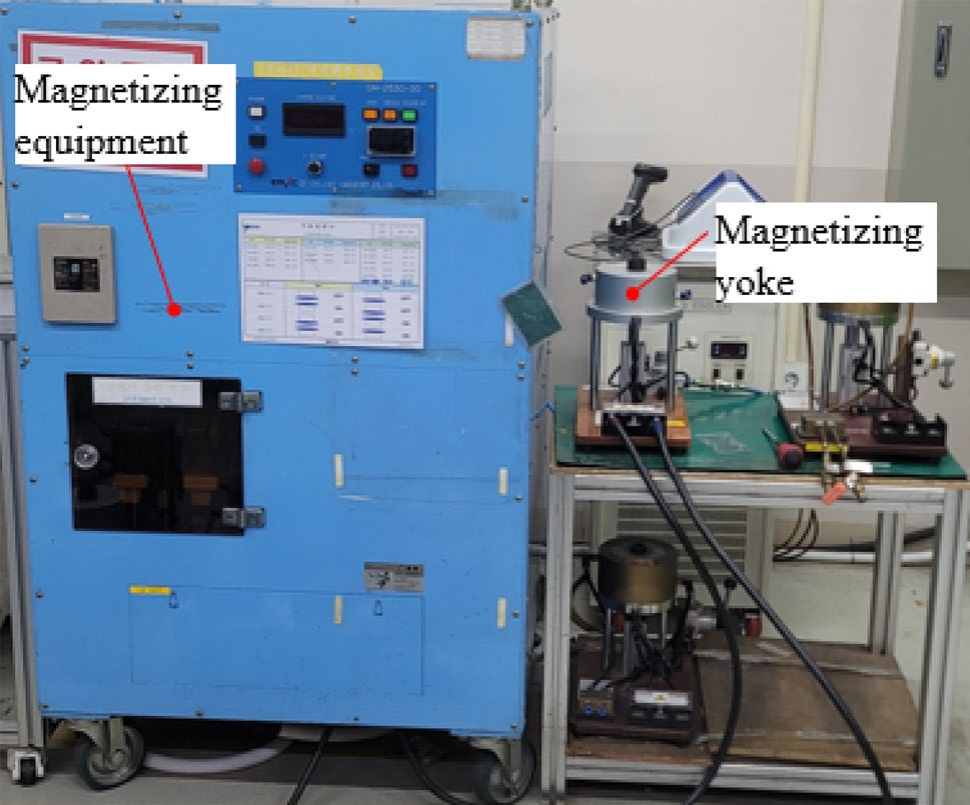
Additional jigs for RTR.

**Figure 15:**
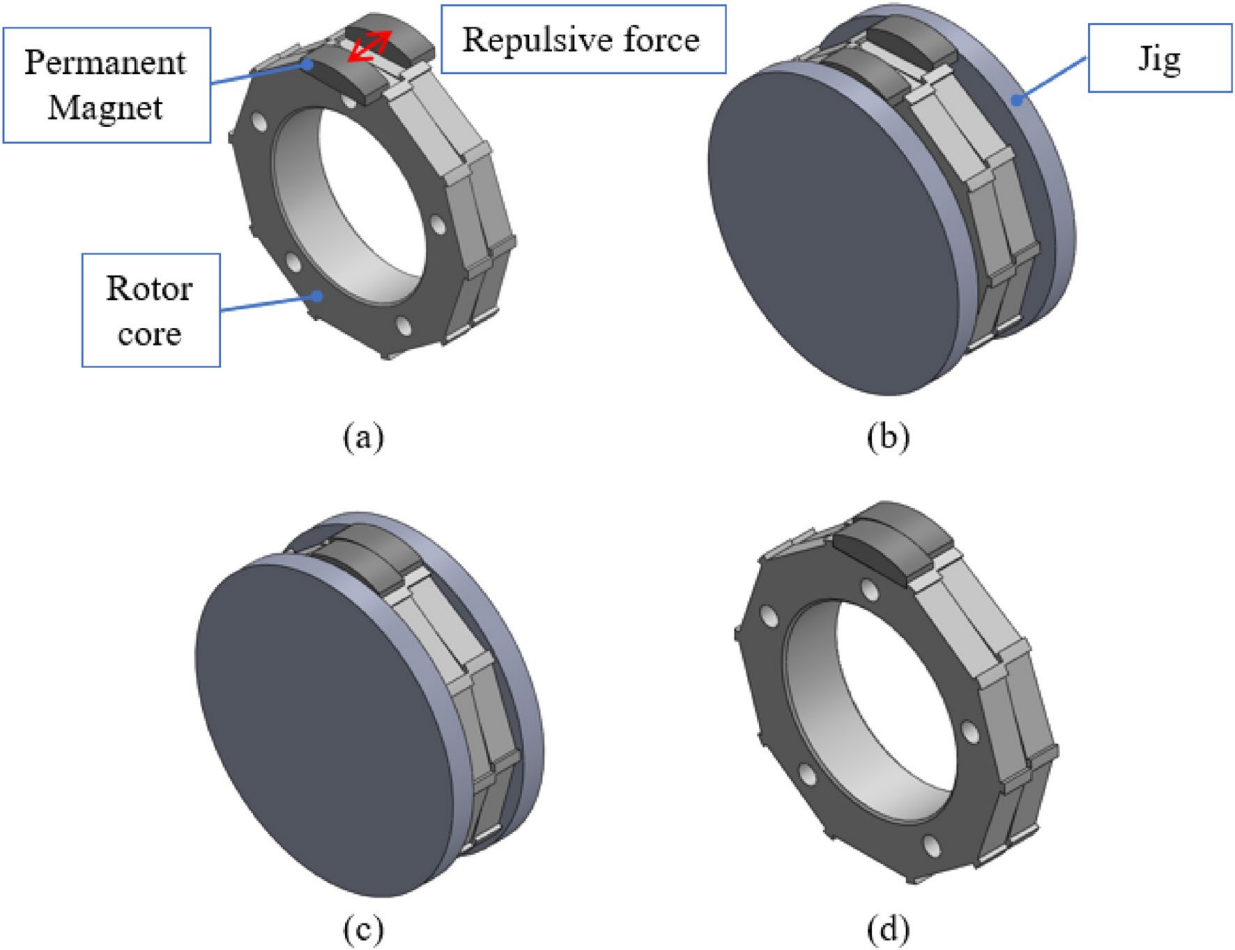
Permanent magnet bonding process for TTR.

**Figure 16:**
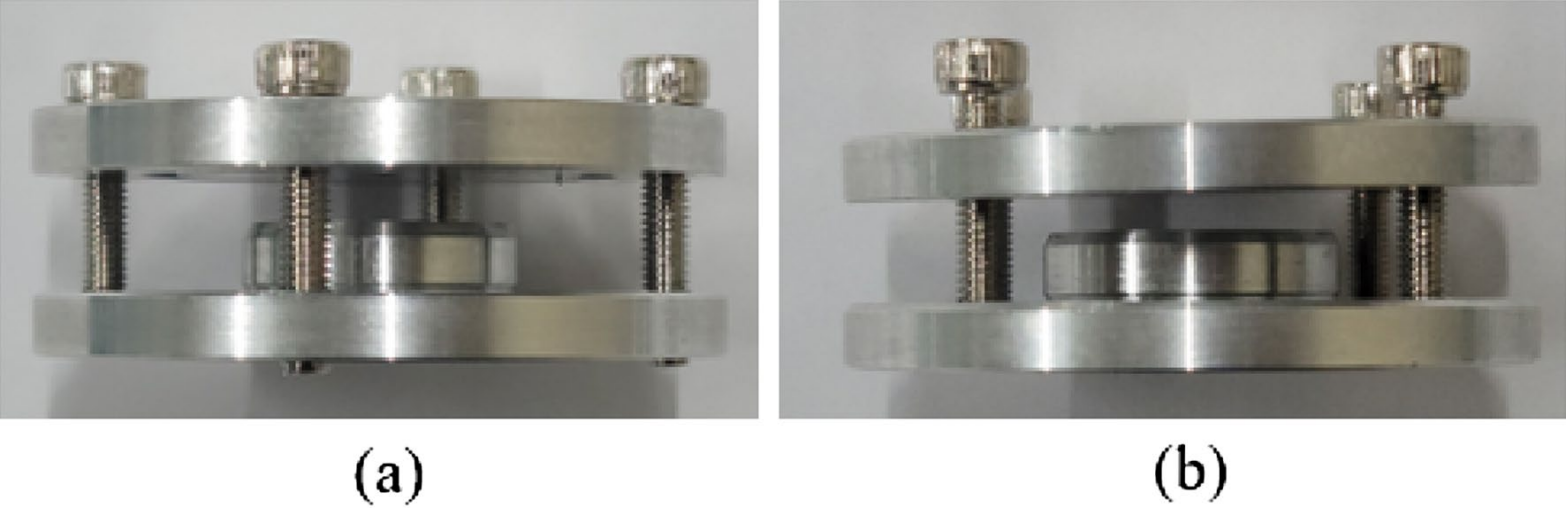
Additional jigs for TTR: (**a**) jig for TTR-I and (**b**) jig for TTR-II.

Specifically, the structural complexity is analyzed in terms of the shape complexity, by counting the number of surfaces to which one pole magnet is attached in the rotor core, and manufacturing complexity, by checking whether additional jigs are necessary. Table 7 lists the evaluations of the structural complexity of the rotors. The RTR’s shape complexity is low, with the permanent magnet of one pole being attached to one surface; however, the manufacturing complexity is high because additional manufacturing jigs are required during magnetization. Figure 14 shows the additional jig, which is the magnetization equipment; magnetization is performed at a high voltage through the magnetization yoke. The TTR’s shape complexity is high, with the permanent magnets per pole being attached to eight surfaces, and the manufacturing complexity is also high because additional manufacturing jigs are required during magnet bonding. Figure 15 shows the permanent magnet bonding process of TTR. Figure 15a shows the state in which the magnets repel each other and do not stick because of the repulsive force. Figure 15b,c show the process by which the magnet is pressed, while bonding using a jig, with a force stronger than the repulsive force of the magnet. Figure 15d is the state in which the rotor is completed by removing the jig after the bond is fixed. Figure 16 shows the additional jigs for TTR; Fig. 16a,b represent TTR-I and TTR-II, respectively. NTR has lower structural complexity, compared to the basic models. NTR’s shape complexity is low, with a magnet of one pole being attached to three surfaces. Similarly, its manufacturing complexity is low because no additional manufacturing jig is required. The shape complexity of NTR is lower than that of the TTR model, and its manufacturing complexity is lower than that of the TTR and RTR models.

**Table 7:** Evaluation of structural complexity of the rotors.

Item	R-SPMSM	T-SPMSM	N-SPMSM
Number of surfaces	1	8	3
Additional jig	O	O	X

## Performance evaluation

In this section, the electromagnetic properties are evaluated through numerical and experimental analyses. Here, R-SPMSM and T-SPMSM are considered as comparison models. The electromagnetic performance is evaluated in terms of the cogging–mutual torque ratio (*γ*) and back-EMF measurements. The cogging–mutual torque ratio, which is inversely proportional to the electromagnetic performance of the SPMSM, is defined as follows.

12 $$\gamma = \frac{{T_{c} }}{{T_{m} }}$$

where ${T}_{c}$ and ${T}_{m}$ are the cogging torque and mutual torque, respectively. The larger the mutual torque and the smaller the cogging torque, the better the performance; therefore, the smaller the cogging–mutual torque ratio, the better the performance of the SPMSM.

### Numerical analysis

The electromagnetic characteristics are analyzed in terms of the cogging torque, mutual torque, and back-EMF. Numerical evaluations of the cogging torque and mutual torque of R-SPMSM-I, T-SPMSM-I, N-SPMSM-I, R-SPMSM-II, T-SPMSM-II, and N-SPMSM-II are performed and the results are compared. In addition, the back-EMF values of the SPMSMs are also measured. The JMAG material conditions are as follows: copper is used for the coil, 35JN230 for the stator core, S45C for the rotor core, NMX-K35SR for the RTR permanent magnet, NMX-S45SH for the TTR permanent magnet, and NMX-43SH for the NTR permanent magnet.

Table 8 presents the numerical analysis results. N-SPMSM-I has a cogging–mutual torque ratio of 0.010%, which is the similar to that of R-SPMSM-I (0.003%) and T-SPMSM-I (0.051%). N-SPMSM-II has a cogging–mutual torque ratio of 0.038%, which is the similar to that of R-SPMSM-II (0.002%) and T-SPMSM-II (0.077%). N-SPMSM-I exhibits a back-EMF of 5.05 V_rms_, which is similar to that of R-SPMSM-II (4.40 V_rms_) and T-SPMSM-II (5.05 V_rms_). N-SPMSM-II exhibits a back-EMF of 7.20 V_rms_, which is similar to that of R-SPMSM-II (6.25 V_rms_) and T-SPMSM-II (7.22 V_rms_).

**Table 8:** Numerical results of electromagnetic performance.

Item	Unit	R-SPMSM-I	T-SPMSM-I	N-SPMSM-I	R-SPMSM-II	T-SPMSM-II	N-SPMSM-II
Cogging torque	mNm	0.01	0.33	0.06	0.02	0.99	0.49
Mutual torque	mNm	547	637	637	1,090	1,279	1,276
Ratio	%	0.003	0.051	0.010	0.002	0.077	0.038
Back-EMF	V_rms_	4.40	5.05	5.05	6.25	7.22	7.20

### Experimental analysis

Experimental evaluations of the cogging torque and mutual torque of R-SPMSM-I, T-SPMSM-I, N-SPMSM-I, R-SPMSM-II, T-SPMSM-II, and N-SPMSM-II are performed and the results are compared. Additionally, the back-EMF values of the SPMSMs are measured. The materials used in the prototype are: copper for the coil, silicon steel plate for the stator core, and stainless steel for the rotor core.

**Figure 17:**
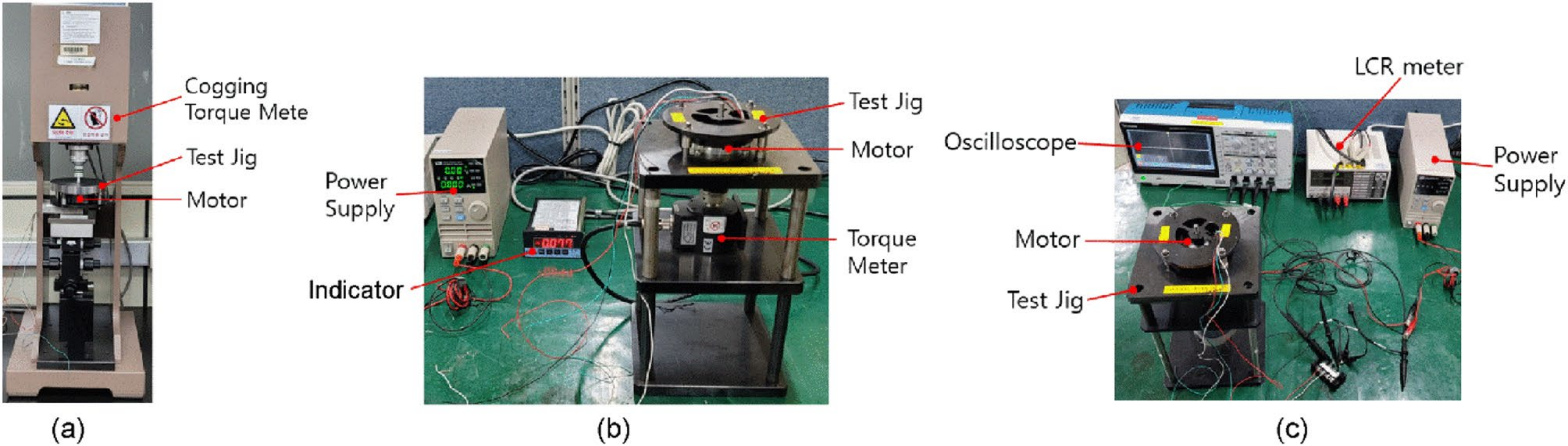
Test bed for measuring (**a**) cogging torque, (**b**) mutual torque, and (**c**) back-EMF.

Figure 17a shows the test bed for measuring the cogging torque. The experimental equipment consists of a cogging-torque analyzer unit, test jig unit, and motor unit. The cogging torque analyzer uses a 50 mNm torque detector, and the test jig part is used to connect the motor and cogging-torque analyzer. The cogging torque is measured by rotating the motor through 360°. The measured cogging torque through equipment, the bearing friction can be included. Since the analysis of cogging torque don’t include the bearing friction, the comparison with analysis and experiment must be done carefully, taking into account various factors such as bearing friction.

Figure 17b shows the test bed for measuring the mutual torque. The test device consists of a DC power supply, an indicator, a torque transducer, jig, and motor. Ictech's product IT6721 is used as the DC power supply to supply current to the motor, and SETech's product YD-3533 is used as an indicator to display the measured torque supplied to a torque meter. SETech's product YDSA(S)-20KC is used as a torque transducer to measure the torque of the motor through the coupling and jig. During the test, the (+) power supply is connected to SPMSM’s U power line and the (−) power supply is connected to SPMSM's V and W power lines. Subsequently, the current is applied to determine the position of the rotor. At this time, a coupling is fastened to connect the shaft of the motor and torque meter. Following the connection of the (+) power supply to the SPMSM's V power line and (−) power supply to the SPMSM's W power line, 5 A_dc_ and 8 A_dc_ are applied to SPMSM-I and SPMSM-II, respectively, to validate the torque displayed on the indicator.

Figure 17c shows the test bed for measuring the back EMF of the motor. The test device consists of an oscilloscope, an LCR meter, a power supply, drive motor, motor, and jig. Tektronix's TBS2000B is used as the oscilloscope and HIOKI's 3511-50 is used as the LCR meter. Ictech's IT6721 is used as the DC power supply, and SPG's S9D90-90D is used as the drive motor. During the test, the resistance and inductance are first measured using the LCR meter to ensure that the motor is functioning normally. Next, the drive motor is connected to the motor through a test jig. The power lines of the SPMSM are connected to the oscilloscope. 20 V_dc_ is applied to the drive motor through the power supply for rotating the SPMSM. At this time, the oscilloscope's measured frequency and back EMF values in the U, V, and W phases are converted to a 1000 RPM basis.

Table 9 lists the experimental results of the electromagnetic performance. N-SPMSM-I's cogging–mutual torque ratio is 0.96%, which is similar to the values of 0.77% and 0.89% of R-SPMSM-I and T-SPMSM-I, respectively. N-SPMSM-II's cogging–mutual torque ratio is 0.83%, which is similar to the values of 0.78% and 0.71% of R-SPMSM-II and T-SPMSM-II, respectively. The back EMF of N-SPMSM-I is 9.18 V_rms_, which is not inferior to the values of 7.65 V_rms_ and 9.02 V_rms_ of R-SPMSM-I and T-SPMSM-I, respectively. The back EMF of N-SPMSM-I is 13.26 V_rms_, which is again not inferior to the values of 10.59 V_rms_ and 12.87 V_rms_ of R-SPMSM-II and T-SPMSM-II, respectively. Because the segmented magnets have a relatively large magnetic flux, the mutual torque is relatively large; nevertheless, the cogging torque is small because of the newly proposed magnet shape and arrangement.

**Table 9:** Experimental results of electromagnetic performance.

Item	Unit	R-SPMSM-I	T-SPMSM-I	N-SPMSM-I	R-SPMSM-II	T-SPMSM-II	N-SPMSM-II
Cogging torque	mNm	4.19	5.79	6.34	7.91	8.84	10.56
Mutual torque	mNm	540	650	660	1010	1240	1270
Ratio	%	0.77	0.89	0.96	0.78	0.71	0.83
Back-EMF	V_rms_	7.65	9.02	9.18	10.59	12.87	13.26

## Conclusions

This study proposed an N-SPMSM with low structural complexity without the loss of electromagnetic performance. Structural, numerical, and experimental analyses were performed and the results were compared to that of basic models. The structural complexity of each rotor was analyzed from the shape and manufacturing perspectives. The electromagnetic characteristics were analyzed from the cogging torque, mutual torque, and back-EMF perspectives. The structural complexity of NTR was confirmed to be lower than that of RTR and TTR. The electromagnetic performance of N-SPMSM turned out to be similar to that of the basic models. The results of this study can act as basic data that can be used for making structural changes to the SPMSMs, in the future. In particular, they can help reduce the cost and time of manufacturing of the SPMSMs by reducing the structural complexity while minimizing the degradation of electromagnetic performance by optimizing factors such as the cogging torque, mutual torque, and back EMF.

## Data Availability

The datasets used and/or analyzed during the current study available from the corresponding author on reasonable request.
